# Tobacco Use among Health Care Workers in Southwestern Saudi Arabia

**DOI:** 10.1155/2013/960292

**Published:** 2013-08-26

**Authors:** Ahmed A. Mahfouz, Abdullah S. Shatoor, Badr R. Al-Ghamdi, Mervat A. Hassanein, Shamsun Nahar, Aesha Farheen, Inasse I. Gaballah, Amani Mohamed, Faten M. Rabie

**Affiliations:** ^1^Department of Family & Community Medicine, College of Medicine, King Khalid University, Abha 61421, Saudi Arabia; ^2^Department of Internal Medicine, Cardiology, College of Medicine, King Khalid University, Abha 61421, Saudi Arabia; ^3^Department of Chest Disease, College of Medicine, King Khalid University, Abha 61421, Saudi Arabia

## Abstract

The present study targeted health care workers (HCWs) in Governmental Hospitals and Primary Health Care Centers in Abha City, southwestern Saudi Arabia. An anonymous self-report questionnaire was used to assess tobacco use and the reasons for smoking. The present study included 736 HCWs. The overall prevalence of tobacco use amounted to 26.3% (14.8% current and 11.5% former users). In a binary logistic regression analysis, males were found significantly more prone to smoke compared to females (aOR = 3.081, 95% CI: 2.004–4.739). Similarly, parental history of tobacco use was found to be a significant risk factor (aOR = 1.540, 95% CI: 1.040–2.278). Among current users, 89.9% were interested in quitting and 66.1% tried before to quit. The prevalence of smoking among HCWs in the present study, besides being a public health problem, represents a potential barrier in involving this group as a first line for tobacco control. There is a need for a national intervention programme in the country in a tailored manner for HCWs to control tobacco use parallel to the running national program for public. These interventions should begin early in basic medical education and to be applied continually during one's medical career.

## 1. Introduction

World Health Organization (WHO) stated that Tobacco use continues to be the leading global cause of preventable death killing nearly 6 million people and causes hundreds of billions of dollars of economic damage worldwide each year. Most of these deaths occur in low- and middle-income countries, and this disparity is expected to widen further over the next several decades. If current trends continue, WHO anticipated that by 2030 tobacco will kill more than 8 million people worldwide each year, with 80% of these premature deaths among people living in low- and middle-income countries. Over the course of the 21st century, tobacco use could kill a billion people or more unless urgent action is taken [1].

The global tobacco youth survey which is a school-based survey conducted to collect data from school students 13–15 years old by using a standardized methodology showed a prevalence of 6.7% of cigarette use and 11.9% of other tobacco use in Saudi Arabia [2]. A recent review article showed more alarming figures. The article reviewed the literature on the epidemiology, consumption, trade, control, prevention, and treatment of tobacco smoking in Saudi Arabia. The prevalence of current smoking in Saudi Arabia ranged in different studies from 2.4 to 52.3% (median = 17.5%). Among school students, the prevalence of current smoking ranges from 12 to 29.8% (median = 16.5%), among university students from 2.4 to 37% (median = 13.5%), and among adults from 11.6 to 52.3% (median = 22.6%). In elderly people, the prevalence of current smoking is 25%. The prevalence of smoking in males ranges from 13 to 38% (median = 26.5%), while in females it ranges from 1 to 16% (median = 9%) [3].

In 2001, on World No Tobacco Day, the Late King Fahd announced that Mecca and Medina would become smoke-free cities. This directive, although not a formal law, has a similar force within the Kingdom of Saudi Arabia. Since then it has adopted a religiously inspired policy approach to strengthen tobacco control. Working within this faith-based paradigm, a National Tobacco Control Programme that focuses on primary prevention and supporting tobacco cessation has been adopted. National legislation also bans smoking in health and educational facilities and on public transport. Health care workers including physicians play an important role in the identification, assessment, and treatment of smokers. Most people regard physicians as the most reliable source of knowledge and advice on matters of health [4]. A physician's own smoking status appears to be a critically important determinant of how their patient's tobacco use is addressed [5]. The degree, to which many health care professionals continue to be smokers themselves, despite obvious knowledge of the consequences of smoking, may undermine global and local efforts to assist smokers to quit using specific clinical interventions.

Abha city is the capital of Aseer region. The region is located in the southwest of Saudi Arabia and lies few kilometers from the northern border of the neighboring Yemen. The objective of the present work is to study tobacco use and assessing related factors among health care workers in Abha city, southwestern Saudi Arabia.

## 2. Materials and Methods

The present study targeted health care workers in all Governmental Hospitals and Primary Health Care Centers (PHCCs) in Abha City. The Study Hospitals included Abha General Hospital, Abha Hospital of Psychiatry, and Aseer Central Hospital. The study PHCCs were Manhal, Wasat-Abha, Numais, Mansak, Azizia, Zera, Kabel, and Hay-Al-Moazafeen PHCCs.

Data were collected through an anonymous self-administered questionnaire survey (distributed during field visits to hospitals and PHCCs), obtaining information on sociodemographic background including age, gender, and professional category. The questionnaire (attached) collected data on characteristics and circumstances of smoking habit including quantity, circumstances, and reason for smoking and quitting potentials. The questionnaire was based on a WHO questionnaire and the related literature on the topic [6–9]. This survey was approved by the Ethical Committee of King Khalid University.

Data were collected during 2010 by level 8 male and female fourth-year medical students and directly supervised by the Family and Community Medicine staff. Data were coded, validated, and analyzed using SPSS software package. Univariate analysis methods were used at 5% level of significance. Binary logistic regression analysis was performed to identify potential risk factors for tobacco use among health care workers including age, gender, nationality, place of work, degrees, place of birth, and parental smoking.

## 3. Results

### 3.1. Description of the Study Sample

 The present study included 736 health care workers (out of 843 questionnaires distributed giving a response rate of 87.3%, no statistical significant differences in response rate were found among hospitals and Primary Health Care Centers HCWs). Out of them, 566 (76.9%) were working in hospitals and the rest (23.1%, 170) from primary health care centers. They were 405 males and 331 females. The sample included 235 nurses (31.9%), 371 physicians (50.4%) working in hospitals, 85 PHC physicians (11.5%), and 45 other health care workers (6.2%). Saudi HCWs represented 43.5% (320) of the sample, followed by Indians (20.9%, 154), Egyptians (14.1, 104), and Sudanese (7.5%, 55).

### 3.2. Prevalence of Tobacco Use and Determinants

**Figure 1:**
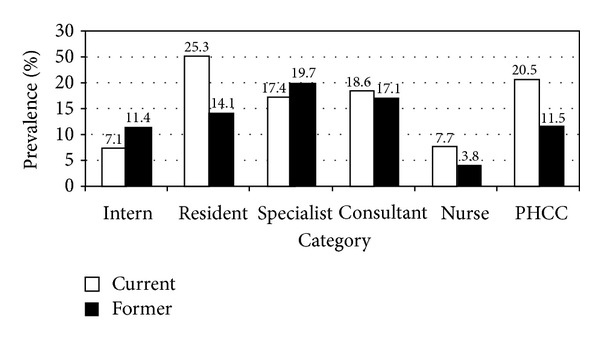
Prevalence of current and former tobacco use among different categories of health care workers in Aseer Region, southwestern Saudi Arabia.

 The overall prevalence of tobacco use among HCWs amounted to 26.3% (14.8% current and 11.5% former users). Among physicians, the figure amounted to 33.6% (18.3% current and 15.3 former users).  Figure 1 shows the prevalence among different categories of HCWs. The highest prevalence of current smokers was among hospital residents (25.3%) followed by PHC physicians (20.5%) and hospital consultants (18.6%). The highest prevalence of former smokers was observed among hospital specialists (19.7%) followed by hospital consultants (17.1%). The overall prevalence of tobacco use among male HCWs amounted to 36.3% (19.3% current and 17.0 former users) compared to 14.2% among females (9.4% current and 4.8 former users).

 In a logistic regression model to identify potential risk factors determining tobacco use among HCWs (Table 1), males were found significantly more prone to smoke compared to females (aOR = 3.081, 95% CI: 2.004–4.739). Similarly, parental history of tobacco use was found to be a significant risk factor (aOR = 1.540, 95% CI: 1.040–2.278).

### 3.3. Practice and Attitudes among Current Tobacco Users

Among current male smokers, 24.4% were occasional smokers compared to 61.3% among females, 25.5% among male smokers smoke less than 5 cigarettes per day compared to 22.6% among females, and those who smoke more than 10 cigarettes per day were 32.1% among males compared to 6.5% among female smokers. The difference is statistically significant (*P* = 0.001). In addition to cigarettes, male smokers used cigars (12.8%) and waterpipe (20.5%).

**Figure 2:**
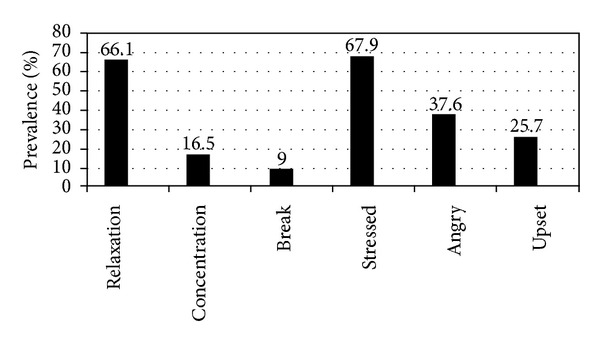
Motives for tobacco use among health care workers smokers in Aseer region, southwestern Saudi Arabia.

The majority of male (96.8%) and female (97.4%) smokers never smoke in front of the patients. The age of starting smoking ranged from 6 to 25 years with an average of 18.2 ± 5.7 years, without any significant gender differences. When asked what you get from smoking (Figure 2), 66.1% mentioned “relaxation,” 16.5% mentioned “more concentration,” and only 9% mentioned “a chance to take a break.” When asked when you smoke, 67.9% mentioned “if stressed,” followed by 37.6% “angry,” and 25.7% “upset.” No significant differences were found by gender.

Regarding intention to quit smoking, 89.9% were interested in quitting smoking and 66.1% tried before to quit. The main motive to stop smoking was health issues (71.9%). The mentioned obstacles in quitting smoking were “feeling tempted on seeing another friend smoking” (38.5%), followed by “feeling personally addicted to the habit of smoking” (28.4%), and “being afraid of the physical consequences of quitting” (21.1%). No statistical significant differences were found in obstacles to quitting by professional grouping or gender.

## 4. Discussion

Health professionals play a crucial role in enhancing tobacco control. As health care providers, they are uniquely positioned to provide patients with information about the harmful effects of tobacco use and assistance in quitting smoking [10]. The prevalence of smoking among HCWs in the present study (26.3%) was similar to that reported for the general adult population in Saudi Arabia (11.6–52.3% with a median of 22.6%) [3]. This situation, besides being a local public health problem, represents a potential barrier in involving this group as a first line for tobacco control.

Twenty years ago, in 1991, physicians in Riyadh, Saudi Arabia, were studied for their smoking habits, and it was found that 48% were smokers and 34% are currently smoking [11]. Ten years later, a study among physicians in Al-Kharj, Saudi Arabia, revealed a prevalence of current tobacco use of 19% [12]. The present study revealed lower figures among physicians (18.3% current and 15.3 former users). The observed decreased trend may be related to the tobacco control law and policies at hospitals. Still, the current figures are alarming figures. The study revealed that one out of each five physicians in Abha City is currently smoker. In other Arab countries, the corresponding figures for current tobacco users were 11.1% among physicians in Oman [7] and 56% among HCWs in Jordan [8]. The corresponding figures of current tobacco users among physicians in other countries were 21.5% in Japan [13], 23% in China [14], 24.9% in Estonia [15], 33.9% in Italy [16], 38.6% in Greece [17], 40% in Bosnia [18], 41% in Turkey [19], and 48.5% in Armenia[20]. The observed difference may be attributed to social and cultural diversities.

The present study revealed that male HCWs are significantly more smokers than females. Similar trend was observed in previous studies in Saudi Arabia [11, 12],Arab countries [7, 8], and other countries [13–20].Cultural factors may explain the gender differences. The WHO Framework Convention for Tobacco Control [1] recognizes “the need for gender-specific tobacco control strategies,” as well as for the “full participation of women at all levels of policy making and implementation of tobacco control measures.” Thus, we need to foster gender-sensitive tobacco prevention intervention programs, starting as early as possible. Women should be always encouraged to take proactive roles in building health educational programs to combat smoking.

The present study showed that parental history of tobacco use was found to be a significant risk factor for tobacco use among HCWs. Similar finding was found among Greek HCWs [17]. The strong association between tobacco use among HCWs and parental history of tobacco use indicates that HCWs who smoke, in some ways, are “victims” of a society in which smoking has a high prevalence; HCWs are therefore left with a deficiency that impedes their important role in the war against smoking. Study of social norms regarding adolescent smoking and their relationship with smoking behavior showed that noticing other teens smoking and the perception that adults care about and disapprove of teen smoking remained significantly related to smoking [21].

The main reasons for smoking in the present study were relaxation and fighting stress, and the cultural misconception of tobacco as a “socializer or helper” under some psychologically stressful conditions is indicated. The latter indicates the influence of cultural and environmental factors as well as personal or individual handicaps. The present study showed the intention of the majority of HCWs to quit smoking. One of the mentioned obstacles was the temptation on seeing a friend smoking. The findings of this study reemphasize the great significance of peer pressure and social gatherings on smoking and also refer to the importance of family members, usually parents, in influencing such behavior. They also remind us of the importance of “role models” in our communities, at home (parents), and at work (managers/chiefs).

World Health Organization in its “Policy Package to Reverse the Tobacco Epidemic” [22] identified two main interventions to facilitate tobacco user cessation in the community. The first is counseling, including face-to-face advice from physicians and other health care workers incorporated into regular medical care as well as over the telephone via quit lines and community programmes. The other is access to low-cost pharmacological therapy. The smoking status of health care workers can have a significant impact on their patients' quit attempts.

There is a need for a national intervention programme in the country in a tailored manner for health care workers to control tobacco use parallel to the running national program. These interventions should begin early in basic medical education and to be applied continually during one's medical career, taking into account different determinants of smoking habits among HCWs.

## Supplementary Material

A copy of the survey instrument was added as Supplementary Material. The anonymous, self-administered survey was distributed during field visits to hospitals and primary health care centers.

## Figures and Tables

**Table 1:** Multivariate analysis, adjusted odds ratio (aOR), and antecedent 95% confidence intervals (CI) of potential risk factors determining smoking habit among health care workers in southwestern Saudi Arabia.

Variable	aOR	95% Confidence interval	
		Upper	Lower
Age group: 30+ years versus less than 30	1.112	0.778	2.625
Gender*: males versus females	**3.081**	**2.004**	**4.739**
Nationality: Saudi versus non-Saudi	1.259	0.824	1.924
Place of work: hospital versus primary health care	1.427	0.884	2.302
Having a postgraduate Degree: yes versus no	0.845	0.514	1.389
Place of birth: city versus village	0.746	0.521	1.067
Parental smoking*: yes versus no	**1.540**	**1.040**	**2.278**

*Significant (*P*< 0.05).
