# Supplementary material for: Tobacco Use among Health Care Workers in Southwestern Saudi Arabia
Source: Biomed Res Int. 2013 Aug 26;2013:960292. doi: 10.1155/2013/960292 (PMC3770036; doi:10.1155/2013/960292)
Supplement: Supplementary file 1 — A copy of the survey instrument was added as Supplementary Material. The anonymous, self-administered survey was distributed during field visits to hospitals and primary health care centers. [file 960292.f1.pdf]

TOBACCO USE AMONG HEALTH CARE WORKERS QUESTIONNAIRE

Dear Colleague,

Smoking is a major public health issue in Saudi Arabia today, and we are very interested in study how HCWs in the region like you **feel towards smoking** and health.

This will take no more than **five minutes** of your time.

Guidelines

This questionnaire is about your **own opinion**. You should provide the answers that *you* think are right. Also, you are *not* expected to know everything. If you feel that you cannot answer a question, just check “I do not know”. You are also free to refuse to answer any question.

Confidentiality

We will keep your responses **strictly confidential**, and the questionnaire is **anonymous**.

Have you ever smoked?

- ☐ NO, I have never smoked — Go to Section B
- ☐ YES, I have smoked — Go to Section A

Are you still smoking?

- ☐ NO, I am a former smoker (have smoked, but currently do not smoke). Go to Section B
- ☐ Yes, I am a regular or occasional smoker — Go To Section A

Section A : Questions for smokers

Please answer the following section if you are a smoker, even an occasional one.

Tobacco use

☐ Cigarettes ☐ Cigars ☐ Water pipe

On average, how many cigarettes do you smoke every day?

☐ I am an occasional smoker who does not smoke every day

☐ 1- 5 ☐ 6 – 10 ☐ More than 10

Do you have other smoking habits?

☐ No I do not ☐ I smoke cigars ☐ I smoke water pipes

Do you regularly smoke in front of your patients? ☐ No ☐ yes

What do you get from smoking?

☐ It relaxes me ☐ It helps me to concentrate

☐ It is an excuse for a break at work

☐ It gives me a confidence **☐ Others:**

Thinking about the reasons or situations why and when you smoke now, please circle the three which most apply to you from the following list, or substitute for your own.

I smoke more when I am:

- ☐Stressed ☐Angry ☐Lonely ☐Bored
- ☐Upset ☐Talking on the telephone ☐Driving
- ☐Relaxing ☐Socializing ☐Thinking ☐Nervous
- ☐Irritable ☐To escape pressure ☐Talking
- ☐Walking ☐After meals ☐Happy

How old were you when you started smoking?................................... years

Why did you start smoking?

- ☐Appear older ☐Appear "cool" ☐Curiosity
- ☐Appear tough/macho ☐Be accepted ☐Rebellion
- ☐Just felt like it ☐Copy friends/family ☐Loneliness
- ☐Feel grown up ☐Out of boredom

How do you feel about quitting smoking?

- ☐I would like to quit smoking ☐I believe I can quit smoking anytime
- ☐I do not like to quit smoking

How interested are you in stopping smoking? (Please check one.)

- ☐Strongly ☐Somewhat ☐not at all

How many times have you tried to stop smoking? (Please check one.)

- ☐Never ☐One ☐Two
- ☐Three ☐Four ☐Five and more

What could motivate you to quit smoking?

☐Health issues ☐The cost of cigarettes ☐Another reason (please specify):………

Which of the following do you consider to be important obstacles to you quitting smoking?

- ☐I find it tempting when I see my family or my friends smoke
- ☐Independently from others, I feel personally addicted to the habit of smoking
- ☐I am afraid of the physical consequences quitting could have on me
- ☐Another obstacle (please specify):………….

Section B

About yourself

Please answer ALL the following questions.

Reminder: all your responses are anonymous and kept confidential.

How old are you? ………………………… Years

Currently you are working in…………………as:…………….

Nationality: ……………

Marital Status:

☐ Single ☐ Married

☐ Widowed ☐ Divorced

Place of Birth:

☐ Village

☐ Small City

☐ Large City

History of Parental Smoking: ☐ Yes ☐ No

Gender: ☐ Male ☐ Female

Thank you for completing this questionnaire.
